# Sensory Processing Difficulties in Children With Eosinophilic Esophagitis

**DOI:** 10.1002/brb3.70642

**Published:** 2025-06-17

**Authors:** Muserrefe Nur Keles, Hacer Ilbilge Ertoy Karagol, Ramazan Yildiz, Odul Egritas Gurkan, Sinan Sari, Bulent Elbasan, Buket Dalgic, Arzu Bakirtas, Gazi University Pediatric Eosinophilic Gastrointestinal Diseases Working Group

**Affiliations:** ^1^ Faculty of Health Sciences, Department of Physiotherapy and Rehabilitation Gazi University Ankara Turkey; ^2^ Faculty of Medicine, Department of Pediatric Allergy Gazi University Ankara Turkey; ^3^ Faculty of Health Sciences, Department of Physiotherapy and Rehabilitation Erzurum Technical University Erzurum Turkey; ^4^ Faculty of Medicine, Department of Pediatric Gastroenterology Gazi University Ankara Turkey

**Keywords:** Oral sensory processing, pediatric eosinophilic esophagitis, sensory integration, sensory profile, vestibular function

## Abstract

**Background:**

Eosinophilic esophagitis (EoE) is a chronic inflammatory esophageal disease associated with dysphagia, food impaction, feeding difficulties, vomiting, and failure to thrive in children. These symptoms result from dysregulated neural control and inflammation‐induced tissue remodeling and may extend beyond esophageal dysfunction to impact sensory processing. This study aimed to evaluate the sensory processing difficulties in children with EoE.

**Methods:**

This prospective case‐control study included children with EoE and age‐matched healthy controls. Sociodemographic data and medical histories were collected. Sensory processing abilities were evaluated using the sensory profile, which evaluates sensory performance across multiple domains.

**Results:**

A total of 69 children aged 3–10 years participated in the study, including 22 diagnosed with EoE and 47 healthy controls. No significant demographic differences were found between groups (*p* > 0.05). Children with EoE exhibited significantly greater sensory processing difficulties in oral sensory processing, vestibular processing, and oral sensory sensitivity compared to healthy controls (*p* < 0.001).

**Conclusions:**

Sensory processing difficulties in children with EoE extend beyond feeding challenges, affecting oral sensory and vestibular processing functions. Incorporating sensory processing assessments into clinical evaluations may improve the understanding of sensory‐related challenges in this population. A multidisciplinary approach could help refine clinical management strategies.

## Graphical abstract

Children with eosinophilic esophagitis demonstrate notable sensory processing difficulties, particularly in oral and vestibular domains. These findings suggest that chronic inflammation may contribute to sensory disruptions through neuroimmune pathways, even in the absence of neurological disease.

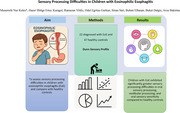

## 1 Introduction

Eosinophilic esophagitis (EoE) is a chronic inflammatory disease of the esophagus, characterized by dysphagia and food impaction in adolescents and adults, as well as feeding difficulties, vomiting, and failure to thrive in infants and young children (Dellon et al. 2018; Markowitz and Clayton 2018). Early recognition of these symptoms is critical, as children at younger ages may be unable to verbally express discomfort. Clinical manifestations are associated not only with inflammation‐induced tissue remodeling but also with disruptions in neural control mechanisms conveyed through afferent vagal pathways (Kim et al. 2024; Mayer 2011; Chrousos 2009). The vagus nerve plays a central role in brain–gut communication by transmitting visceral sensory input from the esophagus to brainstem and limbic structures. In addition, persistent feeding‐related discomfort may activate stress pathways, particularly the hypothalamic–pituitary–adrenal (HPA) axis, which in turn can dysregulate emotional and sensory processing mechanisms in the brain (Kim et al. 2024; Mayer 2011; Chrousos 2009). This neuroimmune interplay may contribute to altered sensory modulation, emotional reactivity, and avoidance behaviors in children with EoE.

According to Ayres’ Sensory Integration Theory, the central nervous system must efficiently organize and process information from multiple sensory modalities—including visual, auditory, tactile, gustatory, olfactory, proprioceptive, vestibular, and interoceptive systems—to generate appropriate motor, behavioral, and emotional responses (Ayres 1972). Sensory development begins prenatally, and early exposure to tactile, vestibular, and proprioceptive input in utero plays a foundational role in shaping neural pathways (Stein and Rowland 2011). Disruptions in one system can affect the functioning of others, resulting in difficulties with postural control, coordination, and adaptive behavior (Blanche and Gunter 2020; Dunn 1997). These challenges may ultimately interfere with a child's ability to participate in daily activities and acquire age‐appropriate developmental skills (Corbett et al. 2016). Therefore, effective sensory integration is essential for healthy neurodevelopment.

Drawing on sensory integration theory, this study hypothesizes that esophageal inflammation in EoE disrupts sensory processing through both neural dysregulation and altered postnatal sensory experiences. Chronic inflammation may increase vagal afferent signaling and central sensitization, contributing to hyper‐responsivity across sensory domains (Kim et al. 2024; Mayer 2011). Moreover, early‐onset symptoms such as vomiting, feeding discomfort, and positional distress may limit sensory exploration during critical developmental windows. These combined factors may help explain the multisensory processing difficulties observed in children with EoE. In addition, previous researches and clinical observations suggest that adaptive eating behaviors commonly observed in these children, such as selective eating, overfilling the mouth, and a preference for different textures, may be linked to underlying sensory processing difficulties (Ertoy Karagol et al. 2021; Yapar et al. 2023; Keles et al. 2023; Koken et al. 2023).

While sensory processing difficulties are documented in feeding disorders, their prevalence in EoE remains unexplored. Sensory processing difficulties have been widely documented in children with neurodevelopmental disorders such as autism spectrum disorder (ASD) and attention‐deficit/hyperactivity disorder (ADHD), where sensory hyper‐responsiveness, oral aversion, and selective eating are common (Türer and Köse 2023; Tran et al. 2022; Pavão and Rocha 2017). Similar profiles have also been observed in children with feeding difficulties. For instance, Davis et al. (2013) found that young children presenting to outpatient feeding clinics displayed significantly elevated sensory sensitivities, particularly in the oral and tactile domains, which were strongly associated with feeding‐related challenges (Davis et al. 2013).

Despite these associations, to our knowledge, no studies to date have specifically examined multidomain sensory integration in this population, either nationally or internationally. Therefore, this study aims to identify and describe the sensory processing characteristics of children with EoE. By identifying sensory integration difficulties in this population, we hope to support the development of more comprehensive and multidisciplinary care approaches tailored to their unique sensory needs.

## 2 Methods

### 2.1 Participants

Approval for conducting this case‐control study with independent groups was per the principles outlined in the Declaration of Helsinki and received ethical approval from the University Ethics Committee (Approval Number: E‐77082166‐604.01.02‐269615). Parents provided written informed consent, and the study was conducted at a University Hospital in Turkey between February 2021 and April 2023. At our center, after the diagnosis of EoE was confirmed by pediatric gastroenterology and pathology departments, the children were followed up by a multidisiplinary team including pediatric gastroenterology, pediatric allergy, pathology, nutrition, and physiotherapy specialists. Diagnosis of EoE was made according to the guideline with symptoms suitable with esophageal dysfunction and esophageal eosinophilia (≥ 15 eosinophil/high‐power field) (Dellon et al. 2018).

According to the G^*^Power program (version 3.1.9.2, Universität Düsseldorf, Germany), a power analysis indicated that a total of 80 participants (40/group) would be required to detect an effect size of 0.80 at a significance level of *p*  =  0.05 with a power of 0.95. However, due to the rarity of EoE in the pediatric population, the actual number of participants included in each group was lower than expected. The EoE group consisted of children aged 3–10 years with an active diagnosis of EoE, confirmed by endoscopic biopsy. The control group included age‐ and sex‐matched children recruited from routine pediatric outpatient clinics for general health follow‐up visits. Exclusion criteria for both groups included the presence of chronic medical conditions such as neurological, rheumatological, or neurodevelopmental disorders; any known psychiatric or developmental diagnoses based on medical records; and unwillingness to participate in the study. Caregivers were required to be the child's primary caregiver, possess adequate cognitive capacity to complete the study forms, and provide informed consent.

### 2.2 Procedure

Following approval from the University Hospital Ethics Committee, data collection was initiated. All children who were newly diagnosed with EoE during the study period and who met the inclusion criteria were invited to participate. Concurrently, children attending routine outpatient follow‐up visits at the general pediatrics clinic, and whose families voluntarily agreed to participate, were recruited as controls. Sociodemographic information, including age, sex, height, and weight, was recorded for all participants. In addition, for children in the EoE group, clinical information related to EoE (e.g., symptom type and severity, age at diagnosis, duration of symptoms) was collected from medical records also, the presence of active EoE symptoms was also recorded, such as dysphagia, food impaction, abdominal pain, vomiting, feeding refusal, chest pain, and heartburn. The sensory profile (SP) was completed by caregivers in a face‐to‐face setting with a physiotherapist who provided guidance as needed.

### 2.3 Measurements

#### 2.3.1 Sensory Profile

The SP is a standardized, 125‐item, caregiver‐reported questionnaire designed to assess sensory processing abilities in children aged 3–10 years (Dunn 1999). It evaluates how sensory processing influences daily functional performance and behavioral responses. The SP is divided into three main sections: sensory processing, modulation, and behavioral and emotional responses.

The sensory processing section consists of six subsections assessing responses to sensory input across different modalities, including auditory, visual, tactile, vestibular, proprioceptive, and oral sensory processing. The modulation section includes five subsections evaluating the ability to regulate sensory input to support functional performance. The behavioral and emotional responses section, with three subsections, examines the impact of sensory processing on emotions and behavior. In addition to section scores, the SP provides nine factor scores that offer insight into specific sensory processing patterns: sensory seeking, emotional reactivity, low endurance/tonus, oral sensory sensitivity, inattention/distractibility, poor registration, sensory sensitivity, sedentary, and fine/perceptive motor coordination (Dunn 1999).

The questionnaire employs a 5‐point Likert scale, where responses range from 1 (*always*) to 5 (*never*). The total score ranges from 125 to 625, with lower scores indicating greater sensory processing difficulties. The scores for the three main sections are calculated by summing the total item scores within each section, whereas the nine factor scores are derived by summing relevant item scores across multiple subsections (Dunn 1999).

The Turkish version of the SP was used in this study, which has been validated and shown to have strong internal consistency across subdomains (Cronbach's alpha values ranging from 0.72 to 0.89) (Kayihan et al. 2015). These values are comparable to those reported in the original English version by Dunn (*α* = 0.75–0.90), supporting the reliability of the adapted tool (Dunn 1999).

For classification, the normal ranges of SP were used in the data analysis (Table 1) (Dunn 1999). Sensory processing patterns were categorized based on standard deviations (SD) from the mean in a typically developing population. A definite difference (DD) was defined as scores ≥ 2 SD from the mean, indicating that the child responds to stimuli significantly more or less than their peers; a probable difference (PD) was defined as scores between 1 and 2 SD, suggesting that the child probably responds to stimuli differently; and a typical performance (TP) was defined as scores within 1 SD of the mean, meaning the child's responses are consistent with those of typically developing peers (Dunn 1999).

**TABLE 1:** Sensory profile classification.

Sensory profile categories	Typical performance (min–max)	Probably difference (min–max)	Definite difference (min–max)
*Sensory processing*			
Auditory processing	30–40	26–29	8–25
Visual processing	32–45	27–31	9–26
Vestibular processing	48–55	45–47	11–44
Touch processing	73–91	65–72	18–64
Multisensory processing	27–35	24–26	8–23
Oral sensory processing	46–60	40–45	12–39
*Sensory modulation*			
Sensory processing related to endurance/tone	39–45	36–38	9–35
Modulation related to body position and movement	41–50	36–40	10–35
Modulation of movement affecting activity level	23–35	19–22	7–18
Modulation of sensory input affecting emotional responses	16–20	14–15	4–13
Modulation of visual input affecting emotional responses and activity level	15–20	12–14	4–11
*Behavioral and emotional responses*			
Emotional/social responses	63–85	55–62	7–54
Behavioral outcomes of the sensory processing	22–30	19–21	6–18
Items indicating thresholds for response	12–15	10–11	3–9
**Sensory profile factors**			
Sensory seeking	63–85	55–62	17–54
Emotional reactivity	57–80	48–56	16–47
Low endurance/tonus	39–45	36–38	9–35
Oral sensory sensitivity	33–45	27–32	9–26
Inattention/distractibility	25–35	22–24	2–21
Poor registration	33–40	30–32	8–29
Sensory sensitivity	16–20	14–15	3–14
Sedentary	12–20	10–11	4–9
Fine/perceptive motor coordination	10–15	8–9	3–7

Abbreviations: Min: minimum; Max: maximum.

### 2.4 Statistical Analysis

All statistical analyses were performed using SPSS version 20.0 (SPSS Inc., Chicago, IL, USA). The normality of continuous variables was assessed visually (using histograms and probability plots) and analytically with the Shapiro–Wilk test. Variables with *p* ≥ 0.05 were considered to be normally distributed. Descriptive statistics were presented as mean ± SD for normally distributed continuous variables and as median (interquartile range) for non‐normally distributed variables. Categorical variables were reported as frequencies and percentages (*n*, %).

Independent samples *t*‐tests were used to compare normally distributed continuous demographic variables between groups. Chi‐square tests or Fisher's exact tests (when expected cell counts were below 5) were used for comparing categorical variables.

Group comparisons of SP classifications were also conducted using Chi‐square tests or Fisher's exact tests, depending on cell counts. To reduce the risk of Type I error due to multiple comparisons, a Bonferroni correction was applied. After correction, statistical significance for sensory processing comparisons was set at *p* ≤ 0.0021. For all other analyses, *p* < 0.05 was considered statistically significant.

Effect sizes were calculated using Cohen's *d* to assess the practical significance of the differences between the EoE and control groups. According to Cohen's guidelines, *d* values of 0.2, 0.5, and 0.8 represent small, medium, and large effect sizes, respectively (Cohen 1988).

## 3 Results

A total of 69 children were included in the study, with 22 diagnosed with EoE and 47 serving as healthy controls. The EoE and control groups were similar in age, sex, height, and weight, with no significant differences between groups (*p* > 0.05) (Table 2 ). Among children with EoE, 17 had active symptoms. Detailed medical histories of children with EoE are presented in Table 2.

**TABLE 2:** Demographic characteristics of participants and disease characteristics of children with EoE.

	Control group (*n* = 47)	EoE group (*n* = 22)	*p*
Age (months) (Mean ± SD)	6.45 ± 1.54	6.45 ± 1.79	0.823^a^
Gender (F/M) *n* (%)	10/12 (45.5/54.5)	23/24 (48.9/51.1)	0.787^b^
Body weight (kg) (Mean ± SD)	22.52 ± 6.67	21.38 ± 7.12	0.285^a^
Height (cm) (Mean ± SD)	117.87 ± 6.45	118.18 ± 13.82	0.645^a^
**Disease characteristics of children with EoE**			
Age of diagnosis (months) Median (IQR)		54.0 (26.0–72.0)	
Age at onset of symptoms (months) Median (IQR)		2.5 (0.0–6.0)	
Duration of symptoms (months) Median (IQR)		61.0 (46.0–75.0)	
Active symptoms, *n* (%)		17 (77.3)^b^	

Abbreviations: EoE: eosinophilic esophagitis; F: female; IQR: interquartile range; M: male; SD: standard deviation.

^a^ Independent samples *t*‐test.

^b^ Chi‐square test.

Distribution and comparison of SP classifications (TP, PD, and DD) between children with EoE and healthy controls are presented in Table 3. Children with EoE demonstrated significantly greater sensory processing difficulties in oral sensory processing (*p* < 0.001, Cohen's *d* = 0.48) and vestibular processing (*p* = 0.002, Cohen's *d* = 0.40), both reflecting medium to large effect sizes. In addition, a significant difference was found in the oral sensory sensitivity subdomain (*p* < 0.001, Cohen's *d* = 0.49), indicating a substantial effect size as well. These findings suggest not only statistical but also clinical relevance.

**TABLE 3:** Distribution and comparison of sensory profile classifications between children with EoE and healthy controls.

Sensory profile categories	Typical performance		Probably difference		Definite difference			
	EoE *n* (%)	Healthy *n* (%)	EoE *n* (%)	Healthy *n* (%)	EoE *n* (%)	Healthy *n* (%)	*p*	Effect size
*Sensory processing*								
Auditory processing	21 (95.4)	43 (91.4)	1 (4.5)	3 (6.4)	—	1 (2.1)	0.748^a^	0.03
Visual processing	21 (95.4)	43 (91.4)	1 (4.5)	3 (6.4)	—	1 (2.1)	0.748^a^	0.03
Vestibular processing	8 (6.3)	41 (87.2)	8 (36.3)	3 (6.4)	6 (27.2)	3 (6.4)	< 0.001^*,^ ^a^	0.39
Touch processing	16 (72.7)	45 (95.7)	3 (13.6)	2 (4.2)	3 (13.6)	—	0.010^a^	0.19
Multisensory processing	20 (91.0)	47 (100)	—	—	2 (9.1)	—	0.098^a^	0.07
Oral sensory processing	8 (6.3)	45 (95.7)	5 (22.7)	1 (2.1)	9 (41.0)	1 (2.1)	< 0.001^*,^ ^a^	0.48
*Sensory modulation*								
Sensory processing related to endurance/tone	17 (77.2)	44 (93.6)	1 (4.5)	1 (2.1)	4 (18.2)	2 (4.2)	0.129^a^	0.13
Modulation related to body position and movement	19 (86.3)	42 (89.3)	2 (9.1)	4 (8.5)	1 (4.5)	1 (2.1)	0.860^a^	0.03
Modulation of movement affecting activity level	22 (100)	44 (93.6)	—	2 (4.2)	—	1 (2.1)	0.480^a^	0.49
Modulation of sensory input affecting emotional responses	22 (100)	44 (93.6)	—	2 (4.2)	—	1 (2.1)	0.480^a^	0.05
Modulation of visual input affecting emotional responses and activity level	22 (100)	43 (91.4)	—	2 (4.2)	—	2 (4.2)	0.370^a^	0.07
*Behavioral and emotional responses*								
Emotional/social responses	19 (86.3)	44 (93.6)	3 (13.6)	3 (6.4)	—	—	0.375^b^	0.06
Behavioral outcomes of the sensory processing	21 (95.4)	44 (93.6)	—	3 (6.4)	1 (4.5)	—	0.170^a^	0.013
Items indicating thresholds for response	22 (100)	47 (100)	—	—	—	—	NA	—
**Sensory profile factors**								
Sensory seeking	20 (91.0)	42 (89.3)	1 (4.5)	5 (10.6)	1 (4.5)	—	0.249^a^	0.01
Emotional reactivity	18 (81.8)	44 (93.6)	4 (18.1)	3 (6.4)	—	—	0.198^b^	0.09
Low endurance/tonus	17 (77.2)	44 (93.6)	1 (4.5)	1 (2.1)	4 (18.2)	2 (4.2)	0.129^a^	0.13
Oral sensory sensitivity	10 (45.4)	45 (95.7)	8 (36.3)	1 (2.1)	4 (18.2)	1 (2.1)	< 0.001^*,^ ^a^	0.40
Inattention/distractibility	22 (100)	44 (93.6)	—	—	—	1 (2.1)	1.000^b^	0.02
Poor registration	19 (86.3)	46 (97.9)	2 (9.1)	1 (2.1)	1 (4.5)	—	0.134^a^	0.09
Sensory sensitivity	16 (72.7)	45 (95.7)	6 (27.2)	2 (4.2)	—	—	0.011^b^	0.18
Sedentary	22 (100)	45 (95.7)	—	2 (4.2)	—	—	1.00^b^	0.03
Fine/perceptive motor coordination	22 (100)	46 (97.9)	—	1 (2.1)	—	—	1.00^b^	0.02

Abbreviations: EoE: eosinophilic esophagitis; NA: not applicable.

^a^ Chi Square test.

^b^ Fisher's Exact test.

*p ≤ 0.0021.

In contrast, no significant differences were observed between the groups in other sensory processing, sensory modulation, or behavioral–emotional response domains (*p* > 0.0021), with most showing negligible to small effect sizes (e.g., auditory processing: Cohen's *d* = 0.03; touch processing: Cohen's *d* = 0.06; emotional reactivity: Cohen's *d* = 0.01). These results indicate that sensory processing challenges in EoE are predominantly centered around oral and vestibular domains, rather than being generalized across all sensory systems.

## 4 Discussion

This is the first study to investigate sensory processing differences in children with EoE, revealing significant challenges not only in oral sensory processing but also in vestibular function. While oral sensory differences were expected due to the direct impact of EoE on feeding and swallowing behaviors, the most striking and previously unexplored finding was the significant vestibular processing differences in this population.

The presence of oral sensory processing differences in children with EoE was expected, given the well‐documented relationship between feeding difficulties and altered oral sensory experiences (Keles et al. 2023; Koken et al. 2023; Mukkada et al. 2010). In our study, 63.6% and 54.5% of children exhibited atypical performance in oral sensory processing and oral sensory sensitivity, respectively. These domains showed medium effect sizes, highlighting that the differences were not only statistically significant but also clinically meaningful. Children with EoE often experience dietary restrictions, discomfort during swallowing, and adaptive eating behaviors, which may contribute to oral hypersensitivity or hyposensitivity (Keles et al. 2023; Koken et al. 2023; Cappellotto and Olsen 2021). Limited exposure to diverse textures and tastes during critical periods of sensory development can lead to altered sensory discrimination, manifesting as food aversions or difficulty detecting oral stimuli. This may, in turn, contribute to compensatory feeding behaviors, such as overstuffing the mouth or preferring extreme taste profiles like very sweet or sour foods (Mukkada et al. 2010; Cappellotto and Olsen 2021). In addition, prolonged esophageal inflammation may disrupt sensory feedback mechanisms, further contributing to atypical oral sensory responses (Sengupta 2006; Brock et al. 2016). Similar patterns have been observed in children with feeding disorders, where sensory deprivation or selective food intake was associated with oral–motor difficulties and atypical sensory integration (Haas 2010; Case‐Smith and Humphry 2005). Given that feeding experiences are a fundamental source of oral sensory input, disruptions in these processes could have long‐term consequences on sensory integration and eating behaviors (Ayres 1972).

The significant differences in vestibular processing observed in children with EoE also suggest that sensory processing challenges in this population may affect broader sensorimotor functions. In our analysis, vestibular processing demonstrated a medium effect size (Cohen's *d* = 0.40), indicating that the observed differences likely have clinical significance, not just statistical relevance. The vestibular system, one of the earliest developing and most dominant sensory systems, plays a central role in integrating postural control, spatial orientation, and motor coordination (Ayres 1972; Cullen 2012). Given its extensive connections with the autonomic nervous system, the vestibular system is also involved in regulating visceral sensory feedback and maintaining homeostatic balance, making it susceptible to immune‐mediated inflammatory processes (Bonaz et al. 2017; Wang et al. 2022).

Although EoE is not a neurological disorder, emerging evidence in other chronic inflammatory gastrointestinal diseases suggests that chronic esophageal inflammation and neuroimmune interactions may influence vestibular function through autonomic dysregulation (Kim et al. 2024; Bonaz et al. 2017; Wang et al. 2022). Inflammatory signaling pathways may affect vestibular nuclei and sensory modulation mechanisms, potentially leading to subtle impairments in balance and postural stability (Kamogashira et al. 2023; Song et al. 2024; Solan and Shelley‐Tremblay 2007). Further research is needed to clarify how neuroimmune alterations in EoE impact vestibular function and sensory‐motor integration.

In addition, early feeding difficulties and gastrointestinal symptoms may inadvertently restrict exposure to movement experiences that are essential for vestibular maturation. Frequent vomiting, discomfort, and feeding aversions can lead to prolonged periods in static positions, such as lying supine or upright to prevent reflux, rather than engaging in varied postural transitions like prone, side‐lying, or tummy time movements that are critical for vestibular system stimulation (Ayres 1972; Witherington et al. 2002). Although motor function was not directly assessed in our study, it is noteworthy that approximately 25% of children with EoE exhibited sensory processing related to endurance/tone and low endurance/tonus subsections with performance in the low endurance/tone subsections falling outside the typical range.

In addition to the observed vestibular differences, another domain that may warrant closer attention is the modulation of movement affecting activity level. Although group differences in this area did not reach statistical significance in our sample, the clinical pattern suggests potential challenges in sensory regulation related to movement in children with EoE. Considering the close interplay between movement modulation and vestibular system functioning, future studies with larger and more diverse samples may yield clearer insights into this sensory domain.

Previous studies have demonstrated that children with neurological and neurodevelopmental disorders exhibit significantly altered sensory processing patterns compared to their typically developing peers (Türer and Köse 2023; Tran et al. 2022; Pavão and Rocha 2017). Beyond our study, only two investigations have examined SPs in children with feeding disorders related to diverse medical diagnoses rather than neurological or developmental conditions (Davis et al. 2013; Smith et al. 2005). The first study found that children with cardiorespiratory and behavioral disorders displayed significantly different SPs compared to those with gastrointestinal disorders, particularly in tactile and taste/smell processing (Davis et al. 2013). The second study reported that children with nonorganic failure‐to‐thrive exhibited differences in oral, vestibular, and tactile sensory processing, along with delayed motor development (Smith et al. 2005). However, the heterogeneity of the samples in both studies made it challenging to isolate the specific impact of gastrointestinal conditions on sensory processing. In contrast, our study focuses on a homogeneous patient population, highlighting the influence of chronic esophageal inflammation on sensory development—particularly in oral and vestibular processing—an aspect not previously explored in the literature.

Importantly, while significant differences emerged in oral sensory processing, oral sensory sensitivity, and vestibular processing, no significant differences were observed in other domains such as auditory, visual, tactile, or proprioceptive processing. This suggests that sensory disruptions in EoE may be domain‐specific, primarily affecting systems related to feeding, motor control, and autonomic function. Given these findings, sensory integration therapy—which has been shown to improve sensory processing, motor coordination, and functional participation in children with neurological and neurodevelopmental conditions (Novak et al. 2022)—may also offer clinical benefits for children with EoE. Although our study did not include an intervention component, structured sensory‐based approaches could potentially enhance feeding behaviors, postural control, and multisensory integration in this population. Future studies with larger samples are needed to determine whether more subtle differences exist in other sensory modalities and to evaluate the effectiveness of such interventions in improving daily functioning and quality of life.

Despite its contributions, this study has several limitations. The relatively small sample size may limit the generalizability of our findings and increase the risk of Type II error. In addition, as in other studies involving children with chronic conditions, the use of caregiver‐reported questionnaires may introduce a potential response bias due to increased parental awareness or concern. Another limitation is the lack of direct motor assessments, which could have provided further insights into the relationship between sensory and motor processing in children with EoE. Furthermore, the cross‐sectional design of the study prevents causal interpretations. It remains unclear whether the observed sensory processing difficulties are a direct result of EoE or whether they may be influenced by additional factors such as dietary restrictions, reduced exposure to diverse sensory experiences during early development, or caregiver‐related stress. These possibilities should be explored in future longitudinal or multimethod studies.

## 5 Conclusion

This study is the first to examine multisensory processing differences in children diagnosed with EoE, revealing significant alterations not only in oral sensory processing, which directly relates to feeding challenges, but also in vestibular functioning, which may impact postural control and sensorimotor integration. These findings suggest that sensory processing difficulties in EoE may extend beyond esophageal symptoms and could affect broader aspects of neurodevelopment.

Given the significant rates of atypical sensory processing observed in this population, we recommend that sensory evaluations—such as the SP used in this study—be considered during the follow‐up of children with EoE who exhibit ongoing feeding difficulties, clumsiness, or behavioral signs of sensory over‐ or under‐responsivity. Incorporating occupational therapists, physiotherapists, speech–language pathologists, and dietitians into the multidisciplinary care team may help identify and address these challenges through appropriate interventions, such as sensory integration therapy, structured feeding programs, and individualized dietary management.

Although current clinical guidelines (Dellon et al. 2018) do not yet include sensory processing assessments as part of routine EoE care, our findings may help lay the groundwork for future multidisciplinary recommendations. Further research is warranted to explore the long‐term impact of sensory differences in this population and to evaluate the effectiveness of sensory‐based interventions tailored to children with EoE.

## Author Contributions

**Muserrefe Nur Keles**: conceptualization, data curation, formal analysis, investigation, methodology, writing – original draft, writing – review and editing. **Hacer Ilbilge Ertoy Karagol**: conceptualization, data curation, investigation, methodology, writing – original draft, writing – review and editing. **Ramazan Yildiz**: conceptualization, data curation, formal analysis, methodology, writing – review and editing. **Odul Egritas Gurkan**: conceptualization, data curation, writing – review and editing. **Sinan Sari**: conceptualization, data curation, writing – review and editing. **Bulent Elbasan**: writing – review and editing, conceptualization. **Buket Dalgic**: conceptualization, data curation, writing – review and editing. **Arzu Bakirtas**: conceptualization, data curation, methodology, investigation, writing – original draft, writing – review and editing.

## Ethics Statement

The study was conducted per the principles outlined in the Declaration of Helsinki and received ethical approval from the Gazi University Ethics Committee (Approval Number: E‐77082166‐604.01.02‐269615). Parents were asked to sign a consent form to allow their children to participate in the study.

## Consent

The authors have nothing to report.

## Conflicts of Interest

The authors declare no conflicts of interest.

## Peer Review

The peer review history for this article is available at https://publons.com/publon/10.1002/brb3.70642

## Data Availability

The data that support the findings of this study are available from the corresponding author upon reasonable request.
